# Association between ABO genotypes and risk of dementia and neuroimaging markers: roles of sex and APOE status

**DOI:** 10.3389/fneur.2024.1391010

**Published:** 2024-05-28

**Authors:** Meiling Li, Ruihong Yu, Xiaoyi Wang, Yanqing Zhao, Qixiang Song, Qi Wang, Chunying Fu, Shiva Raj Mishra, Nipun Shrestha, Salim S. Virani, Dongshan Zhu

**Affiliations:** ^1^Department of Epidemiology, School of Public Health, Cheeloo College of Medicine, Shandong University, Jinan, China; ^2^Department of Disinfection and Sterilization, Pingyin Center for Disease Control and Prevention, Jinan, China; ^3^NHMRC Clinical Trials Center, University of Sydney, Sydney, NSW, Australia; ^4^Westmead Applied Research Centre (WARC), Faculty of Medicine and Health, University of Sydney, Sydney, NSW, Australia; ^5^Section of Global Research, The Aga Khan University, Karachi, Pakistan; ^6^Center for Clinical Epidemiology and Evidence-Based Medicine, Shandong University, Jinan, China

**Keywords:** ABO genotypes, ABO blood-group system, dementia, Alzheimer’s disease, neuroimaging markers

## Abstract

**Background:**

Whether the relationships between ABO blood genotypes (AA, AO, BB, BO, AB, and OO) and dementia are modified by gender and APOE status has been unclear.

**Methods:**

We used data from the UK Biobank, a population-based cohort study of 487,425 individuals. Cox proportional hazards models were used to estimate hazard ratios (HRs) and 95% confidence intervals (CI) between ABO genotypes and risk of dementia. Multivariable linear regression models were used to estimate the relationship between ABO genotypes and MRI-based brain indices.

**Results:**

Overall, 487,425 participants were included at baseline. After 34 million person-years follow up, 7,548 patients developed all-cause dementia. Before stratifying by sex and APOE status, compared to OO genotype, BB genotype was associated with increased risk of all-cause dementia (1.36, 1.03–1.80) and other types dementia (1.65, 1.20–2.28). After stratifying by sex, only in males, BB genotype was associated with higher risk of all-cause dementia (1.44, 1.02–2.09) and other types of dementia (1.95, 1.30–2.93). AB genotype in males was also associated with increased AD (1.34, 1.04–1.72). After further stratifying by APOE e4 status, BB genotype with two APOE e4 alleles showed even stronger association with all-cause dementia 4.29 (1.57, 11.72) and other types dementia (5.49, 1.70–17.69) in males. Also in males, AA genotype with one APOE e4 was associated with increased risks of all-cause dementia (1.27, 1.04–1.55), AD (1.45, 1.09–1.94) and other types dementia (1.40, 1.08–1.81). Linear regression models showed that in both sexes with APOE e4, AA genotype was associated with reduced total grey matter volume.

**Conclusion:**

Sex and APOE e4 carrier status modified the association between ABO genotypes and risk of dementia. In males, BB genotype was consistently associated with increased risk of dementia, especially in those with two APOE e4 alleles. Also, in males with one APOE e4, AA genotype might be linked to higher risk of dementia.

## 1 Introduction

Dementia is a syndrome characterized by the deterioration of cognitive function beyond that expected as a result of biological aging. It manifests as a disturbance of multiple higher cortical functions including frontal executive function, language, memory, attention, visuospatial function and object recognition (1). Global cases with dementia exceeded 50 million in 2018 and will increase to 152 million by 2050 (2). Dementia is caused by many different diseases or injuries that directly or indirectly damage the brain. It encompasses a variety of subtypes. Alzheimer’s disease (AD) may account for 60 to 80% of cases and is the most common primary dementia, followed by vascular dementia (VD), dementia with Lewy bodies (DLB), and frontotemporal dementia (FTD) (3). Also, those who experience the brain changes of multiple types of dementia simultaneously have mixed dementia (4). Dementia has become a major cause of disability, dependency, and death among the elderly, placing a huge burden on patients, their families, and the whole society. The underlying causes of dementia remain complex and debatable. Age-related, genetics, lifestyles, and medical conditions probably interact with the core mechanisms of the dementia (5, 6).

The ABO blood groups are determined by the ABO locus located on chromosome 9 (9q34.1-q34.2) (7). Since the discovery of the ABO blood group system, studies have investigated the relationship between the ABO blood group system and multiple health outcomes, e.g., cardiovascular diseases (CVD) and venous thrombosis (8–12). However, the relationship between ABO blood groups and dementia risk is not well understood, and the findings among previous studies are inconsistent (13, 14). One study using a Scandinavian Record-Linkage database found no association between blood type and dementia (13). Another nested case–control study observed that AB blood group was related to increased risk of cognitive impairment (14). Previous studies mainly focused on the blood groups A, B, AB, and O, but little attention has been paid to the potential accurate ABO genotypes. Besides, no study has examined the relationship between ABO genotypes and brain magnetic resonance imaging (MRI) measures. Evidence has suggested that severe levels of white matter hyperintensities and reduced grey matter volumes were associated with cognitive impairment (15, 16).

The present study aimed to investigate the association between ABO genotypes and risks of all-cause dementia, including Alzheimer’s disease (AD), vascular dementia (VD), and other types of dementia (i.e., non-AD non-VD dementia) in a large-scale prospective cohort study. We also explored the relationship between ABO genotypes and neuroimaging markers of brain health. In addition, the ε4 allele of Apolipoprotein E (APOE e4) is the strongest known genetic risk factor for AD (17, 18). Previous studies have shown that APOE e4 increased risk of amyloid plaque deposition because of its inefficient breakdown of amyloid-β peptides (18, 19). Also, over 60% of persons with AD being female. Dementia rates rise after 60 for both genders, with women’s risk surpassing men’s post-70, likely due to reduced estrogen, which is thought to have a protective effect on the brain (20). We thus also analyzed the effect modification of sex and APOE carrier status in the association between ABO genotypes and risk of dementia.

## 2 Methods

### 2.1 Study participants

The UK Biobank is a large population-based prospective cohort study with over 500,000 participants aged 40–69 years when recruited between 2006 and 2010 in 22 assessment centers throughout the UK. Details of the study design and survey methods have been described elsewhere (21). Briefly, participants were interviewed about socioeconomics, lifestyles, and health information via touchscreen questionnaires and verbal interviews, while blood, urine, and saliva samples were also collected from the assessment centers. All participants gave written informed consent for information and biological data collection. UK Biobank has approval from the North West Multicenter Research Ethics Committee (https://www.ukbiobank.ac.uk/learn-more-about-uk-biobank/about-us/ethics).

In this study, we first excluded participants with missing information on ABO genotype (14,941 individuals were excluded). In the rest, we further excluded those without APOE allele status (16 people were excluded). Last, we further excluded participants with unknown dates of dementia diagnosis (*N* = 7). Finally, data from 487,425 individuals were available to study association of ABO genotypes and dementia and 40,156 participants to study the association of ABO genotypes and MRI-based brain volumes. This research was conducted under UK Biobank application number 68369.

### 2.2 ABO genotypes

Genotyping was performed by Affymetrix using two closely related purpose-designed macro-arrays, BiLEVE and Axiom. These arrays contained 807,411 and 825,927 markers, respectively, and overlapped with 95% common content. Combining results from both arrays, the dataset included 805,426 markers. Genotype imputation was performed to boost the number of markers that can be tested for association. This increased the number of testable variants (22). ABO genotypes of participants were determined based on allele combinations from three single nucleotide polymorphisms (SNPs): rs505922, rs8176746, and rs8176719 in the ABO gene on chromosome 9q34.2 (23). Imputations of blood type from UKB (accessed July 2020), were used in the current study.

### 2.3 Assessment of dementia

We used algorithms for dementia definition developed by the UK Biobank Outcome Adjudication Group (Version date January 2022). The algorithms have identified participants with codes for any cause of dementia and the specific subtypes of AD and VD, through linking to hospital admissions and death registries. We further defined other unspecific types of dementia, using the International Classification of Diseases, Tenth Revision (ICD-10) coding system, including F02, F03, G310, G311, G318, if one or more of these codes were recorded as a primary or secondary diagnosis (Supplementary Table S1).

### 2.4 Neuroimaging markers of brain health

The MRI data were acquired on a Siemens Skyra 3 T scanner (Munich, Germany), including resting-state functional MRI, T1-weighted, diffusion, susceptibility-weighted, and T2-weighted fluid-attenuated inversion recovery images. The details of imaging protocol and processing pipeline have been previously described (24). We used brain imaging-derived phenotypes of total grey matter volume, white matter hyperintensity, and total hippocampal volume, which represent regions related specifically to development of dementia (25–27).

### 2.5 Covariates

Based on previous evidence from existing literature, we included the following factors in analyses as covariates: sex, ethnicity, APOE status, years of education, annual household income level and CVD (28–31). Sex was categorized as male and female. Ethnicity was categorized as white and non-white. APOE e4 carrier status was based on two SNPs: rs7412 and rs429358. APOE status was categorized into three categories of no APOE e4 carrier (APOE e2/e2 or e2/e3 or e3/e3 haplotypes), one APOE e4 carrier (e3/e4 and e2/e4 haplotypes), and two APOE e4 carrier (e4/e4 haplotypes). Years of education was categorized as ≤10, 11–12, >12. Annual household income level was divided into four categories as level 1 (Less than £18,000), level 2 (£18,000 to £30,999), level 3 (£31,000 to £51,999), and level 4 (greater than 52,000). Based on self-reported and clinically diagnosed records of coronary heart disease, stroke and heart failure, participants were divided into those with and without CVD.

### 2.6 Statistical analyses

Characteristics were presented as the median and interquartile range (IQR) for non-normally distributed continuous variables and as percentages (%) for categorical variables. Cox proportional hazards regression models were used to estimate the hazard ratios (HR) and 95% confidence intervals (CI) between ABO genotypes and all-cause dementia, AD, VD, and other types of dementia. People with OO genotype were compared as the reference group. Schoenfeld residuals test was used to examine the violation of proportional hazards (PH) assumption. Schoenfeld residuals showed no correlation with the rank of survival time, indicating no violation of PH assumption. As a person’s ABO genotype is generally fixed for life from birth, participants’ person-years were calculated from the date of birth to the date of reported dementia diagnosis, death, or censored date (January 1, 2022), whichever occurred first. In model 1, no covariate was adjusted; in model 2, sex, ethnicity, and APOE status were adjusted. In model 3, years of education, income level and CVD status were additionally adjusted. As ABO blood groups have been particularly associated with CVD (8–10), and the latter were linked to increased risk of dementia (31), we thus additionally adjusted CVD (a composite of coronary heart disease, angina, heart failure and stroke) in model 3 to evaluate the independent effect of ABO genotypes on dementia risk after accounting CVD risk. Multivariable linear regression models were used to examine the *β* coefficients of ABO genotypes with neuroimaging markers of brain health.

To examine whether sex and APOE status modified the associations between ABO genotypes and dementia, we examined the interaction effect of sex, APOE e4 carrier status and ABO genotypes on risk of dementia by adding a product interaction term to the model (*p* = 0.03 and *p* < 0.01). We then stratified the analyses by sex (female, male) and APOE e4 carrier status. We also performed a mediation analysis to explore the mediating effect of CVD in the association between BB genotype and dementia (Supplementary Table S2). All data were analyzed using SAS version 9.4 (SAS Institute) and the statistical significance was set to *p* value <0.05 at two tails.

## 3 Results

### 3.1 Characteristics of participants

**Figure 1:**
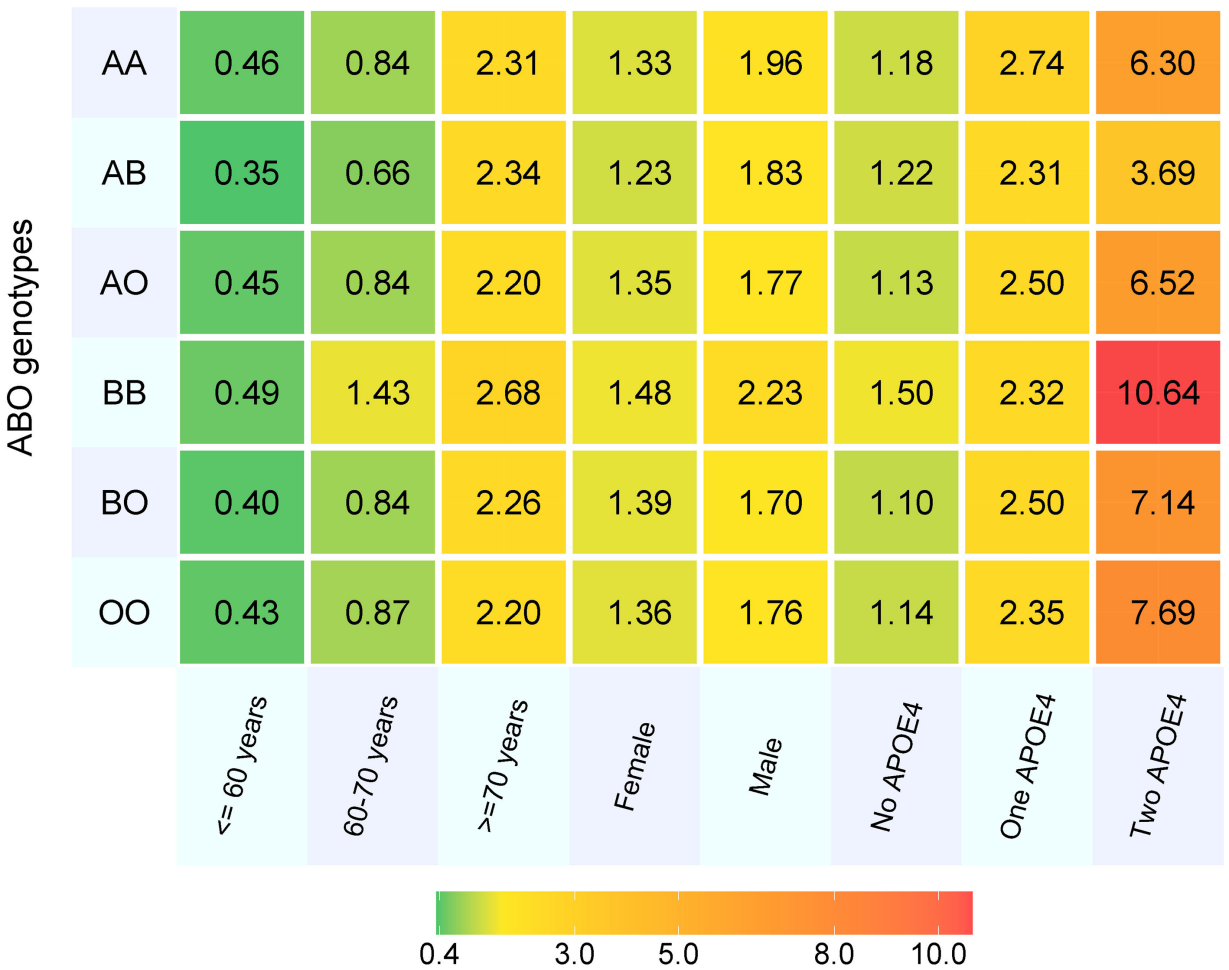
The cumulative incidence of dementia in each ABO genotype by age, sex and APOE4 carrier status. APOE4, Apolipoprotein E ε4 allele.

Totally, 487,425 participants were included at baseline, with mean (SD) age of 56.5 (8.1) years, and 45.8% were men. Over a total of 34,116,799 person-years, we observed 7,548 (1.6%) patients with all-cause dementia, 0.7% patients with AD, 0.3% patients with VD and 0.9% patients with other types of dementia. Participants with BB genotype had highest incidence rate of all-cause dementia (1.8%) (Table 1). After the cumulative incidence of dementia in each ABO genotype was stratified by age, sex and APOE e4 status, people with BB genotype consistently had the highest incidence of dementia except in those with one APOE e4 allele. Also, males had higher incidence than females, and APOE e4 carriers had higher rate than non-carriers (Figure 1).

**Table 1:** Characteristics of participants by ABO genotypes, *N* (%).

Characteristics	ABO genotypes						*N* = 487,425
	AA	AB	AO	BB	BO	OO	
	36,335 (7.5)	17,613 (3.6)	175,200 (35.9)	2,789 (0.6)	44,050 (9.0)	211,438 (43.4)	
Age of last follow-up (years, IQR)	71.0 (64.0–76.0)	71.0 (63.0–76.0)	71.0 (64.0–76.0)	69.0 (62.0–75.0)	71.0 (63.0–76.0)	71.0 (64.0–76.0)	71.0 (64.0–76.0)
**All-cause dementia**							
Yes	588 (1.6)	265 (1.5)	2,703 (1.5)	51 (1.8)	674 (1.5)	3,267 (1.6)	7,548 (1.6)
No	35,747 (98.4)	17,348 (98.5)	172,497 (98.5)	2,738 (98.2)	43,376 (98.5)	208,171 (98.5)	479,877 (98.5)
**Alzheimer’s disease**							
Yes	242 (0.7)	118 (0.7)	1,166 (0.7)	11 (0.4)	271 (0.6)	1,341 (0.6)	3,149 (0.7)
No	36,093 (99.3)	17,495 (99.3)	174,034 (99.3)	2,778 (99.6)	43,779 (99.4)	210,097 (99.4)	484,276 (99.4)
**Vascular dementia**	
Yes	124 (0.3)	52 (0.3)	615 (0.4)	10 (0.4)	136 (0.3)	723 (0.3)	1,660 (0.3)
No	36,211 (99.7)	17,561 (99.7)	174,585 (99.7)	2,779 (99.6)	43,914 (99.7)	210,715 (99.7)	485,765 (99.7)
**Other types of dementia**							
Yes	350 (1.0)	158 (0.9)	1,554 (0.9)	38 (1.4)	392 (0.9)	1923 (0.9)	4,415 (0.9)
No	35,985 (99.0)	17,455 (99.1)	173,646 (99.1)	2,751 (98.6)	43,658 (99.1)	209,515 (99.1)	483,010 (99.1)
**Sex**							
Female	19,666 (54.1)	9,506 (54.0)	95,427 (54.5)	1,491 (53.5)	23,878 (54.2)	114,277 (54.1)	264,245 (54.2)
Male	16,669 (45.9)	8,107 (46.0)	79,773 (45.5)	1,298 (46.5)	20,172 (45.8)	97,161 (46.0)	223,180 (45.8)
**Race**							
White	35,196 (96.9)	15,798 (89.7)	168,522 (96.2)	1828 (65.5)	38,047 (86.4)	199,997 (94.6)	459,388 (94.3)
Non-White	1,139 (3.1)	1815 (10.3)	6,678 (3.8)	961 (34.5)	6,003 (13.6)	11,441 (5.4)	28,037 (5.8)
**Number of APOE4**							
0	27,637 (76.1)	13,453 (76.4)	132,870 (75.8)	2,138 (76.7)	33,449 (75.9)	160,221 (75.8)	369,768 (75.9)
1	8,000 (22.0)	3,808 (21.6)	38,831 (22.2)	604 (21.7)	9,691 (22.0)	46,910 (22.2)	107,844 (22.1)
2	698 (1.9)	352 (2.0)	3,499 (2.0)	47 (1.7)	910 (2.1)	4,307 (2.0)	9,813 (2.0)
**Cardiovascular diseases**							
Yes	6,202 (17.1)	2,854 (16.2)	28,963 (16.5)	495 (17.8)	7,389 (16.8)	34,487 (16.3)	80,390 (16.5)
No	30,133 (82.9)	14,759 (83.8)	146,237 (83.5)	2,294 (82.3)	36,661 (83.2)	176,951 (83.7)	407,035 (83.5)

APOE4, Apolipoprotein E ε4 allele.

### 3.2 ABO genotypes and dementia

Before analyses were stratified by sex and APOE status, compared to people with OO genotype, those with BB genotype had increased risk of all-cause dementia (HR 1.36, 95% CI 1.03–1.80) and other types of dementia (1.65, 1.20–2.28) (Table 2). Mediation analyses showed that 31.4% of the association between BB genotype and all-cause dementia was mediated by CVD (Supplementary Table S2).

**Table 2:** The hazard ratios and 95% confidence intervals (CI) between ABO genotypes and dementia.

Dementia	ABO genotypes					
	AA	AB	AO	BB	BO	OO
**All-cause dementia**						
Model 1	1.04 (0.95, 1.14)	1.03 (0.91, 1.16)	0.99 (0.94, 1.05)	**1.39 (1.05, 1.83)** ^**^	1.04 (0.96, 1.13)	1.00 (ref)
Model 2	1.05 (0.96, 1.15)	1.02 (0.90, 1.16)	1.00 (0.95, 1.05)	**1.37 (1.04, 1.81)** ^*^	1.03 (0.94, 1.12)	1.00 (ref)
Model 3	1.05 (0.96, 1.14)	1.03 (0.91, 1.17)	0.99 (0.94, 1.05)	**1.36 (1.03, 1.80)** ^*^	1.02 (0.93, 1.10)	1.00 (ref)
**Alzheimer’s disease**						
Model 1	1.04 (0.91, 1.20)	1.12 (0.92, 1.35)	1.04 (0.97, 1.13)	0.73 (0.40, 1.32)	1.02 (0.90, 1.16)	1.00 (ref)
Model 2	1.05 (0.92, 1.21)	1.12 (0.93, 1.35)	1.05 (0.97, 1.14)	0.75 (0.41, 1.36)	1.01 (0.89, 1.16)	1.00 (ref)
Model 3	1.05 (0.92, 1.21)	1.14 (0.94, 1.37)	1.04 (0.97, 1.13)	0.75 (0.42, 1.36)	1.02 (0.89, 1.16)	1.00 (ref)
**Vascular dementia**						
Model 1	0.99 (0.82, 1.20)	0.91 (0.69, 1.21)	1.02 (0.92, 1.14)	1.24 (0.66, 2.31)	0.95 (0.79, 1.14)	1.00 (ref)
Model 2	1.00 (0.82, 1.21)	0.91 (0.69, 1.21)	1.03 (0.92, 1.15)	1.23 (0.66, 2.30)	0.94 (0.79, 1.14)	1.00 (ref)
Model 3	1.00 (0.82, 1.21)	0.93 (0.70, 1.23)	1.02 (0.91, 1.13)	1.19 (0.64, 2.22)	0.93 (0.77, 1.11)	1.00 (ref)
**Other types of dementia**						
Model 1	1.05 (0.94, 1.18)	1.04 (0.88, 1.22)	0.97 (0.91, 1.04)	**1.76 (1.28, 2.43)** ^**^	1.03 (0.92, 1.14)	1.00 (ref)
Model 2	1.07 (0.95, 1.19)	1.03 (0.88, 1.21)	0.98 (0.92, 1.05)	**1.66 (1.20, 2.30)** ^**^	1.00 (0.90, 1.12)	1.00 (ref)
Model 3	1.06 (0.95, 1.19)	1.03 (0.88, 1.22)	0.97 (0.91, 1.04)	**1.65 (1.20, 2.28)** ^**^	0.99 (0.89, 1.10)	1.00 (ref)

Model 1: Unadjusted. Model 2: Adjusted for sex (male, female), ethnicity (white, non-white), and APOE status (no APOE4, one APOE4, two APOE4). Model 3: Model 2 + income level (less than 18,000£, 18,000 to 30,999£, 31,000 to 51,999£, Greater than 52,000£), years of education (<=10 years, 11–12 years, >12 years), and cardiovascular diseases including coronary heart disease, stroke, and heart failure (yes, no). ^*^p < 0.05, ^**^ *P* < 0.01.

After the analyses were stratified by sex, no significant association was observed between ABO genotypes and dementia in females. However, in males, BB genotype was linked to elevated risk of all-cause dementia (1.44, 1.02–2.09) and other types of dementia (1.95, 1.30–2.93). Also, in males, AB genotype was associated with increased risk of Alzheimer’s disease, with HRs (95%CI) of 1.34 (1.04, 1.72) (Table 3).

**Table 3:** The hazard ratios and 95% confidence intervals (CI) between ABO genotypes and dementia stratified by sex ^a^.

Sex	ABO genotypes	All-cause dementia	Alzheimer’s disease	Vascular dementia	Other types of dementia
Female					
	AA	0.97 (0.85, 1.11)	0.91 (0.75, 1.12)	0.92 (0.68, 1.25)	1.00 (0.85, 1.19)
	AB	0.96 (0.79, 1.15)	0.96 (0.72, 1.27)	0.97 (0.63, 1.48)	0.92 (0.71, 1.18)
	AO	0.97 (0.90, 1.05)	1.05 (0.94, 1.17)	0.91 (0.77, 1.08)	0.94 (0.86, 1.04)
	BB	1.26 (0.83, 1.93)	0.94 (0.44, 1.97)	1.18 (0.44, 3.17)	1.30 (0.77, 2.21)
	BO	1.04 (0.92, 1.17)	1.00 (0.83, 1.20)	0.98 (0.75, 1.30)	0.98 (0.84, 1.15)
	OO	1.00(ref)	1.00(ref)	1.00(ref)	1.00(ref)
Male					
	AA	1.11 (0.99, 1.25)	1.20 (1.00, 1.45)	1.05 (0.82, 1.35)	1.12 (0.96, 1.31)
	AB	1.10 (0.93, 1.30)	**1.34 (1.04, 1.72)** ^**^	0.90 (0.62, 1.31)	1.14 (0.92, 1.41)
	AO	1.01 (0.94, 1.09)	1.04 (0.93, 1.17)	1.09 (0.95, 1.26)	1.00 (0.91, 1.10)
	BB	**1.44 (1.02, 2.09)** ^*^	0.56 (0.21, 1.49)	1.20 (0.53, 2.69)	**1.95 (1.30, 2.93)** ^**^
	BO	0.99 (0.88, 1.12)	1.03 (0.85, 1.25)	0.88 (0.69, 1.13)	1.00 (0.86, 1.16)
	OO	1.00 (ref)	1.00 (ref)	1.00 (ref)	1.00 (ref)

^a^ HRs were adjusted for ethnicity, APOE status, income, education and cardiovascular diseases (including coronary heart disease, stroke, and heart failure). ^*^p < 0.05, ^**^p < 0.01.

After the analyses were further stratified by APOE e4 carrier status, in males with no APOE e4, BB genotype was linked to increased risk of all-cause dementia (1.59, 1.03–2.46) and other types of dementia (2.03, 1.25–3.31). In males with one APOE e4 allele, AA genotype was associated with elevated risk of all-cause dementia (1.27, 1.04–1.55), AD (1.45, 1.09–1.94) and other types of dementia (1.40, 1.08–1.81). Also, AB (1.75, 1.19–2.58) genotypes was linked to elevated risk of AD in males with one APOE e4. In males with two APOE e4 alleles, BB genotype showed even stronger association with all-cause dementia (4.29, 1.57–11.72) and other types of dementia (5.49, 1.70–17.59) (Table 4).

**Table 4:** The hazard ratios and 95% confidence intervals (CI) between ABO genotypes and dementia stratified by sex and APOE4^a^.

Sex	APOE status	ABO genotypes	All-cause dementia	Alzheimer’s disease	Vascular dementia	Other types of dementia
Female	No APOE e4					
		AA	0.95 (0.80, 1.14)	0.96 (0.72, 1.29)	0.93 (0.63, 1.38)	0.92 (0.73, 1.16)
		AB	1.07 (0.83, 1.37)	1.08 (0.72, 1.62)	1.13 (0.67, 1.91)	1.00 (0.73, 1.37)
		AO	0.93 (0.84, 1.03)	1.02 (0.87, 1.21)	0.88 (0.70, 1.11)	0.92 (0.80, 1.04)
		BB	1.18 (0.65, 2.14)	0.88 (0.28, 2.76)	1.70 (0.54, 5.34)	0.96 (0.43, 2.16)
		BO	1.07 (0.90, 1.26)	1.22 (0.95, 1.58)	0.85 (0.57, 1.27)	0.97 (0.78, 1.20)
		OO	1.00 (ref)	1.00 (ref)	1.00 (ref)	1.00 (ref)
	One APOE e4					
		AA	0.98 (0.79, 1.22)	0.92 (0.67, 1.26)	0.81 (0.47, 1.39)	1.05 (0.79, 1.40)
		AB	0.87 (0.63, 1.20)	1.01 (0.66, 1.55)	0.89 (0.44, 1.83)	0.83 (0.53, 1.29)
		AO	1.04 (0.92, 1.17)	1.09 (0.93, 1.29)	0.89 (0.67, 1.18)	1.00 (0.85, 1.18)
		BB	1.47 (0.78, 2.74)	0.90 (0.29, 2.81)	0.71 (0.10, 5.05)	2.09 (1.04, 4.23)*
		BO	1.08 (0.89, 1.31)	0.91 (0.67, 1.22)	1.09 (0.71, 1.67)	1.09 (0.84, 1.41)
		OO	1.00 (ref)	1.00 (ref)	1.00 (ref)	1.00 (ref)
	Two APOE e4					
		AA	1.02 (0.65, 1.59)	0.70 (0.37, 1.35)	1.43 (0.49, 4.19)	1.48 (0.85, 2.58)
		AB	0.63 (0.31, 1.29)	0.37 (0.12, 1.17)	–	0.68 (0.25, 1.87)
		AO	0.95 (0.75, 1.20)	0.97 (0.72, 1.30)	1.29 (0.71, 2.32)	0.91 (0.65, 1.28)
		BB	0.94 (0.13, 6.86)	1.39 (0.19, 10.21)	–	–
		BO	0.81 (0.54, 1.22)	0.64 (0.37, 1.13)	1.52 (0.65, 3.60)	0.66 (0.35, 1.25)
		OO	1.00 (ref)	1.00 (ref)	1.00 (ref)	1.00 (ref)
Male	No APOE e4					
		AA	1.08 (0.93, 1.27)	1.17 (0.90, 1.52)	1.05 (0.76, 1.45)	1.00 (0.81, 1.23)
		AB	1.16 (0.94, 1.43)	1.27 (0.89, 1.81)	0.98 (0.61, 1.56)	1.23 (0.95, 1.61)
		AO	1.04 (0.95, 1.14)	1.04 (0.89, 1.22)	1.16 (0.97, 1.39)	0.99 (0.88, 1.11)
		BB	1.59 (1.03, 2.46)*	0.49 (0.12, 1.97)	1.15 (0.42, 3.10)	2.03 (1.25, 3.31)**
		BO	0.93 (0.80, 1.09)	0.96 (0.73, 1.26)	0.91 (0.66, 1.25)	0.90 (0.73, 1.10)
		OO	1.00(ref)	1.00(ref)	1.00(ref)	1.00(ref)
	One APOE e4					
		AA	1.27 (1.04, 1.55)*	1.45 (1.09, 1.94)*	1.27 (0.85, 1.89)	1.40 (1.08, 1.81)**
		AB	1.21 (0.90, 1.63)	1.75 (1.19, 2.58)**	0.94 (0.48, 1.86)	1.18 (0.80, 1.75)
		AO	1.06 (0.93, 1.20)	1.16 (0.96, 1.41)	1.08 (0.84, 1.40)	1.06 (0.90, 1.26)
		BB	0.69 (0.26, 1.84)	–	0.77 (0.11, 5.51)	1.18 (0.44, 3.17)
		BO	1.08 (0.88, 1.32)	1.14 (0.84, 1.55)	0.88 (0.56, 1.37)	1.09 (0.84, 1.43)
		OO	1.00 (ref)	1.00 (ref)	1.00 (ref)	1.00 (ref)
	Two APOE e4					
		AA	0.78 (0.50, 1.22)	0.7 (0.36, 1.34)	0.29 (0.07, 1.21)	0.95 (0.54, 1.67)
		AB	0.38 (0.16, 0.92)*	0.61 (0.22, 1.66)	0.31 (0.04, 2.27)	0.28 (0.07, 1.15)
		AO	0.73 (0.57, 0.94)*	0.75 (0.53, 1.06)	0.76 (0.47, 1.25)	0.91 (0.66, 1.26)
		BB	4.29 (1.57, 11.72)**	3.47 (0.83, 14.54)	3.47 (0.46, 26.44)	5.49 (1.70, 17.69)**
		BO	1.11 (0.77, 1.61)	1.09 (0.65, 1.84)	0.79 (0.33, 1.86)	1.39 (0.88, 2.19)
		OO	1.00 (ref)	1.00 (ref)	1.00 (ref)	1.00 (ref)

^a^ HRs were adjusted for ethnicity, income, education, and cardiovascular diseases. * *P* < 0.05, ** *P* < 0.01.

### 3.3 ABO genotypes and neuroimaging markers of brain health

AA genotype was associated with reduced total grey matter volume (*β*: −2397.83, *p* < 0.01), and AB was related to higher total white matter hyperintensities (β: 0.06, *p* = 0.03) (Table 5). After the analyses were stratified by sex and APOE status (Supplementary Table S3), AA genotype was associated with reduced gray matter volume in women (*β*: −7398.10, p < 0.01) and men (*β*: −10567.07, *p* < 0.001) with one APOE e4 allele. It was also found that AA genotype was associated with severe white matter hyperintensities in men carrying one APOE e4 allele (β:0.14, *p* = 0.02). However, in APOE e4 homozygotes of either sex, the AA genotype associated with higher total gray matter volume, indicating an effect modification from APOE status.

**Table 5:** Associations between ABO genotypes and total grey matter, total white matter hyperintensity volume and total hippocampal (mm^3^).

ABO genotypes	Total grey matter		Total white matter hyperintensities		Total hippocampal	
	*β*	*P*	*β*	*P*	*β*	*P*
AA	−2397.83	**0.01**	0.03	0.13	−11.27	0.45
AB	−2117.26	0.09	0.06	**0.03**	−1.63	0.94
AO	208.59	0.68	−0.02	0.05	7.40	0.38
BB	770.94	0.82	0.07	0.37	36.79	0.52
BO	725.15	0.38	−0.02	0.22	3.21	0.82
OO	Ref		Ref		Ref	

All volume measures have been deconfounded for age and age^2 at the imaging visit, a scaling factor for head size and imaging site. White matter hyperintensity volume was log-transformed due to a skew distribution. *β* = unstandardized betas coefficients. The bold values indicated significant *p* values.

## 4 Discussion

### 4.1 Summary of findings

In this large prospective cohort study, we found that BB blood genotype was associated with increased risk of all-cause dementia and other types of dementia. Also, sex and APOE carrier status modified the association between ABO genotypes and dementia. In addition, AA genotype was associated with reduced grey matter volume and AB genotype was related with higher white matter hyperintensities volume.

### 4.2 Comparison with other studies

There were few studies on the relationship between ABO genotypes and cognitive impairment, and the findings were inconsistent (13, 14). In elderly patients undergoing unilateral total hip arthroplasty surgery, Li et al. found that elderly patients with type A blood had higher risk of developing postoperative cognitive dysfunction than those with type O blood (21.4% vs. 10.7%, *p* < 0.01) (32). In a nested case–control study involving of 495 cases with cognitive impairment and 587 controls, Alexander et al. found that those with blood group AB had an increased risk of cognitive impairment (odds ratio (OR) 1.82, 95%CI 1.15–2.90), with age, race, region, and sex being adjusted. After hemostatic factor was further adjusted, the OR for group AB was attenuated to 1.42 (0.78, 2.61) (14). A Scandinavian Record-Linkage Study including blood donors from Sweden and Denmark observed a decreased risk of all-cause dementia (rate ratio (RR) 0.95, 95%CI 0.90–0.99) in people with blood group A, compared to those with blood group O (13). Nevertheless, this study defined ABO blood group using blood types (four phenotypes) rather than genotypes (six genotypes) and the association between genotypes (e.g., AO and AA) and dementia cannot be examined (13). The inconsistent findings between previous studies might be explained by low statistical power due to limited sample size, unrepresentative samples like blood donors, variations in blood-group system measurement, and residual confounders unadjusted, such as APOE e4. Also, previous studies have not further analyzed the interaction between APOE allele status and gender in the relationship between blood type and dementia. It is possible that individuals with certain genotypes may have a higher risk of dementia.

In our study, adopting ABO genotypes, BB genotype was suggested to be associated with an increased risk of all-cause dementia and other types of dementia, especially in males. Also, sex was a potential effect modifier in the association between AA and AB genotypes and AD, i.e., in males, AA (a boundary significance) and AB genotype were linked to increased risk of AD. These findings were also supported by the association of AA and AB genotypes with MRI-based brain volumes which showed that AA was related to decreased volume of total grey matter and AB was possibly linked to increased total white matter hyperintensities, a neuroimaging indicator of dementia (15). WMH may elevate dementia risk by impairing cortex-subcortical nuclei connections (15). Studies suggest WMH are linked to slower information processing and executive dysfunction due to myelin or axonal loss (15, 33). Additionally, WMH could hinder extravascular protein drainage and lead to amyloid-β accumulation (34). The stable associations with other types of dementia indicated that ABO genotypes (especially BB) may play a role in ageing-related neurodegenerative disorders besides of AD and VD. Further experimental or clinical studies are needed to elucidate the association between ABO genotypes and degenerative neurological disorders and the underlying pathogenic mechanisms. An intriguing finding was that in males with two APOE e4 alleles, AB and AO genotypes were linked to lower risk of all-cause dementia. The mechanisms of interactions between AB, AO genotypes and APOE genotype warrant a further investigation.

### 4.3 Potential mechanisms

Although the underlying mechanism of relationship between ABO genotypes and dementia remains to be further elucidated, the possible etiologies may involve both neurodegenerative and vascular pathways. Evidence has suggested that BB genotype individuals had lower expression levels of the GDNF family receptor alpha-3 protein (9), which was associated with axon regeneration and improved treatments in neurodegenerative diseases (35). In addition, a single SNP at the ABO locus was highly correlated with sICAM-1 concentration, suggesting that histo-blood group antigens may affect neuroinflammatory adhesion processes. On the other hand, numerous studies showed that non-O blood type individuals had an increased risk of CVD and thrombosis through its effects on hemostatic factors, such as FVIII/vWF (7, 10). The “heart-to-brain” connection explains that cardiovascular injuries lead to cerebral hypoperfusion (36), which contributes to the formation of tau-containing neurofibrillary tangles and amyloid β (Aβ) plaques (37). In our analyses, the size of the association between ABO genotypes (e.g., BB genotype) and dementia risk was attenuated slightly after the further adjustment of CVD. Mediation analyses also showed that the mediating effect of CVD in the association between BB genotype and dementia was around 30%. This indicated that there was direct effect between ABO genotypes and dementia risk. More mechanistic studies are needed to further elucidate the potential pathways through which blood genotypes play a role in dementia.

### 4.4 Strengths and limitations

To the best of our knowledge, this might be the first study to evaluate the association between ABO genotypes and all-cause and cause specific dementia and to examine their relationships with neuroimaging markers of brain health. The large sample size also enabled us to stratify analyses by sex and APOE e4 carrier status. Also, dementia was ascertained from primary care, hospital admissions and mortality data records, avoiding bias from self-reported data. Dementia outcomes have been validated in previous studies. One study demonstrating positive predictive value (PPV) for all-cause dementia were 80–87% (38). We also acknowledged several limitations of our study. First, we did not investigate Rh-status and FUT2, which may determine the secretor status of ABO (39). Second, incident dementia was not always well captured through hospital inpatient records and death registries. Some mildly cognitive impairment people may not go to the hospital, resulting that dementia cases were likely to be underreported. Third, the sample size of the BB genotype in our main analysis was relatively small compared with the OO genotype. Nevertheless, the distribution of ABO blood group in European was in line with previous studies (40). In addition, there might be some residual confounders unmeasured or unknown variables that drive the relationship between ABO genotypes and dementia. Last, the majority of participants in our study were of European descent, the generalization of our results to other populations should be interpreted with caution.

## 5 Conclusion

BB blood genotype was associated with increased risk of all-cause dementia and other types of dementia. The associations between ABO genotypes and dementia were moderated by sex and APOE status. AA genotype might be related to raised risk of Alzheimer’s disease in males with one APOE e4 allele.

Our findings contribute to the growing body of evidence suggesting that genetics play a role in the risk of developing dementia. The study indicates that certain ABO genotypes, especially BB in males and specific APOE e4 statuses, link to higher dementia risks. This insight could lead to personalized early detection and prevention in high-risk individuals based on their genetic profiles. Integrating ABO and APOE genetic testing into public health screenings might help identify those at increased dementia risk, especially in populations with prevalent risk-associated genotypes.

## Data availability statement

Publicly available datasets were analyzed in this study. This data can be found at: https://www.ukbiobank.ac.uk/.

## Ethics statement

The studies involving humans were approved by the UK National Health Service’s National Research Ethics Service (ref 11/NW/0382). The studies were conducted in accordance with the local legislation and institutional requirements. The participants provided their written informed consent to participate in this study.

## Author contributions

ML: Conceptualization, Formal analysis, Methodology, Writing – original draft. RY: Writing – review & editing. XW: Writing – review & editing. YZ: Writing – review & editing. QS: Writing – review & editing. WQ: Data curation, Writing – review & editing. CF: Writing – review & editing. SM: Writing – review & editing. NS: Writing – review & editing. SV: Writing – review & editing. DZ: Conceptualization, Methodology, Supervision, Writing – review & editing.
