# Supplementary material for: Association between ABO genotypes and risk of dementia and neuroimaging markers: roles of sex and APOE status
Source: Front Neurol. 2024 May 28;15:1391010. doi: 10.3389/fneur.2024.1391010 (PMC11165032; doi:10.3389/fneur.2024.1391010)
Supplement: Supplementary file 1 [file Data_Sheet_1.DOCX]

***Supplementary Material***

**Supplementary Table 1:** Code list for Dementia and the Major Diagnostic Pathologies in UK Biobank

**Supplementary Table 2:** The mediating effect of CVD in the association between BB genotype and dementia

**Supplementary Table 3:** Associations between ABO genotypes and total grey matter volume, total white matter hyperintensity volume and total hippocampal volume stratified by sex and APOE (mm^3^)

| Supplementary Table 1. Code list for Dementia and the Major Diagnostic Pathologies in UK Biobank | | | | | | | | | | | | | | |
| --- | --- | --- | --- | --- | --- | --- | --- | --- | --- | --- | --- | --- | --- | --- |
| Code Type | | Code | | Code Description | | AD | | VD | | Other Types of Dementia | | All Cause Dementia | |  |
| UK Biobank  Self Report | | Field 20002  Code 1263 | | Dementia/Alzheimers/Cognitive Impairment | | 0 | | 0 | | 0 | | 1 | |  |
| ICD 9 | | 290.2 | | Senile dementia, depressed or paranoid type | | 0 | | 0 | | 0 | | 1 | |  |
| ICD 9 | | 290.3 | | Senile dementia with acute confusional state | | 0 | | 0 | | 0 | | 1 | |  |
| ICD 9 | | 290.4 | | Arteriosclerotic dementia | | 0 | | 1 | | 0 | | 1 | |  |
| ICD 9 | | 291.2 | | Other alcoholic dementia | | 0 | | 0 | | 0 | | 1 | |  |
| ICD 9 | | 294.1 | | Dementia in other conditions classified elsewhere | | 0 | | 0 | | 0 | | 1 | |  |
| ICD 9 | | 331.0 | | Alzheimer's disease | | 1 | | 0 | | 0 | | 1 | |  |
| ICD 9 | | 331.1 | | Pick's disease | | 0 | | 0 | | 0 | | 1 | |  |
| ICD 9 | | 331.2 | | Senile degeneration of brain | | 0 | | 0 | | 0 | | 1 | |  |
| ICD 9 | | 331.5 | | Creutzfeldt-Jakob disease | | 0 | | 0 | | 0 | | 1 | |  |
| ICD 10 | | A81.0 | | Sporadic Creutzfeldt-Jakob disease | | 0 | | 0 | | 0 | | 1 | |  |
| ICD 10 | | F00 | | Dementia in Alzheimer's disease | | 1 | | 0 | | 0 | | 1 | |  |
| ICD 10 | | F00.0 | | Dementia in Alzheimer's disease with early onset | | 1 | | 0 | | 0 | | 1 | |  |
| ICD 10 | | F00.1 | | Dementia in Alzheimer's disease with late onset | | 1 | | 0 | | 0 | | 1 | |  |
| ICD 10 | | F00.2 | | Dementia in Alzheimer's disease, atypical or mixed type | | 1 | | 0 | | 0 | | 1 | |  |
| ICD 10 | | F00.9 | | Dementia in Alzheimer's disease, unspecified | | 1 | | 0 | | 0 | | 1 | |  |
| ICD 10 | | F01 | | Vascular dementia | | 0 | | 1 | | 0 | | 1 | |  |
| ICD 10 | | F01.0 | | Vascular dementia of acute onset | | 0 | | 1 | | 0 | | 1 | |  |
| ICD 10 | | F01.1 | | Multi-infarct dementia | | 0 | | 1 | | 0 | | 1 | |  |
| ICD 10 | | F01.2 | | Subcortical vascular dementia | | 0 | | 1 | | 0 | | 1 | |  |
| ICD 10 | | F01.3 | | Mixed cortical and sub-cortical vascular dementia | | 0 | | 1 | | 0 | | 1 | |  |
| ICD 10 | | F01.8 | | Other vascular dementia | | 0 | | 1 | | 0 | | 1 | |  |
| ICD 10 | | F01.9 | | Vascular dementia, unspecified | | 0 | | 1 | | 0 | | 1 | |  |
| ICD 10 | | F02 | | Dementia in other diseases classified elsewhere | | 0 | | 0 | | 1 | | 1 | |  |
| ICD 10 | | F02.0 | | Dementia in Picks disease | | 0 | | 0 | | 1 | | 1 | |  |
| ICD 10 | | F02.1 | | Dementia in Creutzfeldt-Jacob disease | | 0 | | 0 | | 1 | | 1 | |  |
| ICD 10 | | F02.2 | | Dementia in Huntington’s disease | | 0 | | 0 | | 1 | | 1 | |  |
| ICD 10 | | F02.3 | | Dementia in Parkinson’s disease | | 0 | | 0 | | 1 | | 1 | |  |
| ICD 10 | | F02.4 | | Dementia in HIV disease | | 0 | | 0 | | 1 | | 1 | |  |
| ICD 10 | | F02.8 | | Dementia in other specified diseases classified elsewhere | | 0 | | 0 | | 1 | | 1 | |  |
| ICD 10 | | F03 | | Unspecified dementia | | 0 | | 0 | | 1 | | 1 | |  |
| ICD 10 | | F05.1 | | Delirium superimposed on dementia | | 0 | | 0 | | 0 | | 1 | |  |
| ICD 10 | | F10.6 | | Mental and behavioural disorders due to use of alcohol - amnesic syndrome | | 0 | | 0 | | 0 | | 1 | |  |
| ICD 10 | | G30 | | Alzheimer’s disease | | 1 | | 0 | | 0 | | 1 | |  |
| ICD 10 | | G30.0 | | Alzheimer’s disease with early onset | | 1 | | 0 | | 0 | | 1 | |  |
| ICD 10 | | G30.1 | | Alzheimer’s disease with late onset | | 1 | | 0 | | 0 | | 1 | |  |
| ICD 10 | | G30.8 | | Other Alzheimer's disease | | 1 | | 0 | | 0 | | 1 | |  |
| ICD 10 | | G30.9 | | Alzheimer's disease unspecified | | 1 | | 0 | | 0 | | 1 | |  |
| ICD 10 | | G31.0 | | Circumscribed brain atrophy | | 0 | | 0 | | 1 | | 1 | |  |
| ICD 10 | | G31.1 | | Senile degeneration of brain | | 0 | | 0 | | 1 | | 1 | |  |
| ICD 10 | | G31.8 | | Other specified degenerative diseases of nervous system | | 0 | | 0 | | 1 | | 1 | |  |
| ICD 10 | | I67.3 | | Binswanger's disease | | 0 | | 1 | | 0 | | 1 | |  |

| Supplementary Table 2. The mediating effect of CVD in the association between BB genotype and dementia | | | | | | | |
| --- | --- | --- | --- | --- | --- | --- | --- |
| Mediation Path | Total effect |  | Direct effect | |  | Indirect effect | |
|  | Size |  | Size | % |  | Size | % |
| BB→ CVD→ All cause dementia | 0.43 |  | 0.30 | 68.6 |  | 0.14 | 31.4 |
| BB→ CVD→ Other types of dementia | 0.64 |  | 0.50 | 77.6 |  | 0.14 | 22.4 |

| Supplementary Table 3. Associations between ABO genotypes and total grey matter volume, total white matter hyperintensity volume and total hippocampal volume stratified by sex and APOE (mm^3^) | | | | | | | | | | |  |
| --- | --- | --- | --- | --- | --- | --- | --- | --- | --- | --- | --- |
| Sex | APOE status | ABO genotypes | total grey matter volume | |  | total white matter hyperintensities volume | |  | total hippocampal volume | | |
|  |  |  | β | P |  | β | P |  | β | P | |
| Female | No APOE e4 | |  |  |  |  |  |  |  |  | |
|  |  | AA | 176.08 | 0.90 |  | 0.01 | 0.79 |  | 5.55 | 0.80 | |
|  |  | AB | -1869.71 | 0.37 |  | 0.02 | 0.70 |  | -23.16 | 0.48 | |
|  |  | AO | 762.28 | 0.34 |  | -0.04 | **0.01** |  | -4.12 | 0.74 | |
|  |  | BB | -5460.44 | 0.32 |  | 0.03 | 0.82 |  | -64.66 | 0.45 | |
|  |  | BO | -234.41 | 0.86 |  | -0.04 | 0.19 |  | -21.71 | 0.29 | |
|  |  | OO | ref |  |  | ref |  |  | ref |  | |
|  | One APOE e4 | |  |  |  |  |  |  |  |  | |
|  |  | AA | -7398.10 | **<0.01** |  | 0.01 | 0.84 |  | 32.27 | 0.43 | |
|  |  | AB | -588.37 | 0.88 |  | 0.07 | 0.41 |  | -56.23 | 0.34 | |
|  |  | AO | -437.95 | 0.77 |  | -0.02 | 0.48 |  | -9.23 | 0.69 | |
|  |  | BB | -11103.24 | 0.33 |  | 0.18 | 0.47 |  | 65.24 | 0.71 | |
|  |  | BO | 4165.55 | 0.09 |  | -0.02 | 0.76 |  | 26.75 | 0.49 | |
|  |  | OO | ref |  |  | ref |  |  | ref |  | |
|  | Two APOE e4 | |  |  |  |  |  |  |  |  | |
|  |  | AA | 20560.69 | **0.04** |  | 0.05 | 0.81 |  | 51.11 | 0.73 | |
|  |  | AB | -7790.90 | 0.61 |  | 0.24 | 0.43 |  | 412.28 | 0.07 | |
|  |  | AO | 5082.60 | 0.34 |  | -0.03 | 0.80 |  | 64.18 | 0.43 | |
|  |  | BB | 40581.80 | 0.24 |  | 0.18 | 0.79 |  | 49.81 | 0.92 | |
|  |  | BO | -1922.60 | 0.83 |  | 0.10 | 0.59 |  | -69.10 | 0.62 | |
|  |  | OO | ref |  |  | ref |  |  | ref |  | |
| Male | No APOE e4 | |  |  |  |  |  |  |  |  | |
|  |  | AA | -2475.85 | 0.09 |  | 0.03 | 0.35 |  | -31.27 | 0.23 | |
|  |  | AB | -1623.70 | 0.42 |  | 0.06 | 0.18 |  | 48.67 | 0.18 | |
|  |  | AO | -50.70 | 0.95 |  | -0.02 | 0.39 |  | 19.41 | 0.19 | |
|  |  | BB | 4162.73 | 0.43 |  | 0.11 | 0.34 |  | 106.92 | 0.26 | |
|  |  | BO | 2295.11 | 0.09 |  | -0.03 | 0.26 |  | 31.85 | 0.20 | |
|  |  | OO | ref |  |  | ref |  |  | ref |  | |
|  | One APOE e4 | |  |  |  |  |  |  |  |  | |
|  |  | AA | -10567.07 | **<0.001** |  | 0.14 | **0.02** |  | -78.18 | 0.11 | |
|  |  | AB | -5689.80 | 0.14 |  | 0.16 | 0.06 |  | -88.23 | 0.22 | |
|  |  | AO | -985.70 | 0.52 |  | 0.04 | 0.25 |  | 13.26 | 0.64 | |
|  |  | BB | 7902.75 | 0.45 |  | 0.08 | 0.74 |  | 100.46 | 0.61 | |
|  |  | BO | -4752.35 | 0.05 |  | 0.05 | 0.32 |  | -36.34 | 0.43 | |
|  |  | OO | ref |  |  | ref |  |  | ref |  | |
|  | Two APOE e4 | |  |  |  |  |  |  |  |  | |
|  |  | AA | 27333.23 | **0.01** |  | -0.09 | 0.72 |  | 162.66 | 0.42 | |
|  |  | AB | 1541.13 | 0.89 |  | 0.39 | 0.13 |  | 54.59 | 0.80 | |
|  |  | AO | 1529.26 | 0.79 |  | 0.08 | 0.55 |  | 92.06 | 0.38 | |
|  |  | BB^*^ | **-** | **-** |  | **-** | **-** |  | **-** | **-** | |
|  |  | BO | 177.32 | 0.98 |  | 0.02 | 0.90 |  | 77.34 | 0.64 | |
|  |  | OO | ref |  |  | ref |  |  | ref |  | |

*As there was only one male participant having BB genotype and carried two APOE e4 alleles, we were unable to obtain accurate regression coefficients because the sample size was too small.
